# Two-year outcomes of *Faith in Action/Fe en Acción*: a randomized controlled trial of physical activity promotion in Latinas

**DOI:** 10.1186/s12966-022-01329-6

**Published:** 2022-07-30

**Authors:** Elva M. Arredondo, Jessica Haughton, Guadalupe X. Ayala, Donald Slymen, James F. Sallis, Lilian G. Perez, Natalicio Serrano, Sherry Ryan, Rodrigo Valdivia, Nanette V. Lopez, John P. Elder

**Affiliations:** 1 Department of Psychology, San Diego State University and the Institute for Behavioral and Community Health, 9245 Sky Park Ct, San Diego, CA 92123 USA; 2 Institute for Behavioral and Community Health, San Diego State University, San Diego, USA; 3 School of Public Health, San Diego State University and the Institute for Behavioral and Community Health, San Diego, USA; 4 School of Public Health, San Diego State University, San Diego, USA; 5 Herbert Wertheim School of Public Health and Human Longevity Science, University of California San Diego, La Jolla, USA; 6 Australian Catholic University, Melbourne, Australia; 7 Behavioral and Policy Sciences Department, RAND Corporation, Santa Monica, USA; 8 School of Public Health, University of Illinois at Chicago, Chicago, USA; 9 School of Public Affairs, San Diego State University, San Diego, USA; 10 The Roman Catholic Diocese of San Diego, San Diego, USA; 11 Health Sciences, Northern Arizona University, Flagstaff, USA

**Keywords:** Faith based intervention, Community health worker, Hispanic/Latinos, Health promotion, Exercise, Health equity

## Abstract

**Background:**

Latina women are less likely to report engaging in leisure-time physical activity (PA) than non-Latina white women. This study evaluated the 24-month impact of a faith-based PA intervention targeting Latinas.

**Methods:**

The study is a cluster randomized controlled trial of a PA intervention or cancer screening comparison condition, with churches as the randomization unit. A total of 436 Latinas (aged 18-65 years) from 16 churches who engaged in low levels of self-report and accelerometer-based PA were enrolled. The experimental condition was a 24-month PA intervention, with in-person classes, social support, and environmental changes, led by community health workers (i.e., *promotoras*). At baseline, 12-, and 24 months, we assessed changes in accelerometer-based and self-reported moderate to vigorous physical activity (MVPA; primary outcomes). Secondary outcomes were light intensity activity, sedentary time, body mass index (BMI), and waist circumference.

**Results:**

After adjusting for sociodemographic factors, a mixed effects analysis found significant increases in self-reported leisure time MVPA (*p* < 0.005) and marginal increases in accelerometer-assessed MVPA (*p* < 0.08) 24 months post-baseline in the intervention compared to the attention-control condition. Data showed significant associations between PA class attendance and engaging in MVPA as assessed by self-report and accelerometry. No significant changes were found for light activity, sedentary time, BMI, or waist circumference.

**Conclusions:**

Participants who attended the PA classes at least once a month engaged in significantly higher MVPA compared to those who did not. Maximizing engagement and maintenance strategies to enhance PA maintenance could contribute to important long-term health benefits.

**Trial registration:**

NCT01776632, Registered March 18, 2011.

**Supplementary Information:**

The online version contains supplementary material available at 10.1186/s12966-022-01329-6.

## Background

National guidelines recommend that adults engage in aerobic activity at least 150 min per week at moderate-to vigorous-intensity and perform muscle strengthening activities on 2 or more days per week [1]. Despite public health efforts to promote physical activity (PA), 80% of Americans do not meet these guidelines, with only half achieving the aerobic activity guideline and fewer than one-third achieving the muscle strengthening guideline [1]. Large disparities in PA exist, notably in Latina women, with only about one third meeting the aerobic activity guideline [2]. Inadequate PA is associated with increased risk of obesity [3], metabolic syndrome [4], cardiovascular disease [5], and cancers [4]. Considering Latinas are at greater risk of these conditions compared to non-Hispanic Whites [6, 7], addressing low PA may help reduce these health disparities. Promoting moderate to vigorous PA (MVPA) among individuals with low PA has been shown to have the greatest health benefits [8, 9], but the majority of intervention studies have focused on short-term effectiveness [10].

Research that examines the effectiveness of long-term interventions (i.e., 1-year or more of intervention activities) on participants’ PA is limited. Most interventions with Latina women have been of short duration (6 months or less) with self-reported PA as the primary outcome [11–13]. Interventions that facilitate and support PA over longer periods of time are likely to have a bigger impact on individuals’ health outcomes than interventions that support PA in the short term [14]. Longer duration interventions in Latinas (9 months or longer) have also typically used self-reported PA as the primary outcome [15–17] and have been frequently home-based, using telephone counseling or mailed newsletters [18].

Community-based interventions that use a neighborhood community center or other convenient facility as a gathering place may promote greater PA engagement and maintenance among community members [17, 19]. Faith-based organizations such as churches are an ideal setting for lifestyle interventions and provide an opportunity to engage participants for a longer duration intervention. A systematic review by Parra and colleagues found that interventions delivered in faith-based organizations increased PA and positively influenced measures of health and fitness in participants [20]. The supportive environment of the church offers many advantages that might increase the effectiveness of an intervention. These include an alignment with the mission of the church for promoting physical, emotional, and spiritual health; familiarity and historical presence of the church within the community; lower costs of using church-owned facilities for PA programs; and social support from fellow parishioners and clergy. Faith-based programs show promise for United States (US) Latinos, due to the large proportion who attend services weekly (40% for Catholics; 71% for Protestants). Most US Latinos identify as Catholic (55%) or Protestant (22%) (Pew Research Center, 2014).

This study describes the PA changes in the *Faith in Action* study (*Fe en Acción*), a church-based clustered randomized trial to promote PA in Latinas [21]. *Faith in Action* focused on Latina women because they are a fast growing female racial/ethnic minority group in the US and engage in lower levels of leisure-time PA than Latino men [22], thereby increasing their risk for many chronic diseases. *Promotoras* (i.e., community health workers) delivered the intervention, which followed an ecological framework that targeted potential mediators at multiple levels including individual, interpersonal, organizational, and environmental. This report examines PA over 24-months and builds on our 12-month analysis of *Faith in Action* that found significant increases in accelerometer-assessed and self-reported MVPA [23]. In the current study, we examined the hypothesis that a multilevel PA intervention will increase PA among Latinas compared to the comparison condition across 24 months, thus examining the long term of impact of *Faith in Action*. This analysis also examined the impact of the intervention on participants’ light intensity activity, sedentary behavior, body mass index (BMI), and waist circumference. The protocol was approved by the San Diego State University Institutional Review Board.

## Methods

*Faith in Action* was a clustered randomized controlled trial (two-arm parallel assignment) involving 16 Catholic churches (*n* = 436 Latinas) in San Diego County. Based on pilot data, we assumed an intraclass correlation of 0.05 with an alpha level of 0.05. The power achievable with 16 churches and 20 participants per church was estimated at 85% as determined by accelerometer-assessed MVPA. When considering anticipated drop-out rates of 25%, our target sample size was 432 participants. The Catholic Diocese of San Diego provided a list of churches, and those that had at least 200 US Latino families and one Spanish-language service per week were invited to participate in the 2-year intervention with assessments at baseline, 12 months, and 24 months. Churches were stratified by size and randomly assigned to a PA promotion intervention or an attention-control condition (cancer screening comparison condition described below) by a statistician who did not have any knowledge of the church. In each participating church, 2-3 *promotoras* were recruited from the target community, hired, and underwent 6 weeks of training by the Project Manager and Physical Activity Specialist to implement the program according to their experimental condition. The *promotora*s did not have prior training in leading PA programs.

Recruitment and evaluation staff were blind to each church’s experimental condition during participant recruitment and measurement activities. Participants were recruited via church announcements, word of mouth, flyers, and printed materials (e.g., church bulletins) over 3 months. To be eligible, women had to self-identify as Latina, be between the ages of 18 and 65, attend the participating church at least four times a month for any reason, plan on attending the church for the next 24 months, live within 15 minutes driving distance of the church, not attend other churches enrolled in the study, not have a health condition that would preclude them from being physically active, and report low PA and engage in less than 250 minutes/week of MVPA as assessed by accelerometry during screening. Further details about the study design, measures, and full study protocol are published elsewhere [21].

### Physical activity intervention

*Faith in Action* intervention activities were offered free at the participant’s church or local parks and community centers. The intervention targeted multiple levels of influence on PA (individual, interpersonal, organizational, environmental), as described in greater detail elsewhere [23, 24], and is included in National Cancer Institute’s Evidence-Based Cancer Control Programs (EBCCP): database (https://ebccp.cancercontrol.cancer.gov/index.do).

Briefly, *Faith in Action* was informed by preliminary research and a church-based pilot study [25, 26]. Each week over 24 months, *promotoras* led six weekly classes in each church (cardio dance, strength training, and walking groups) scheduled at times to accommodate participants’ schedules and occurring both indoors and outdoors, at the church site (e.g., halls, meeting rooms, and parking lots), and in the community (e.g., parks, recreation centers, and trails). PA classes were programmed as follows: a welcoming prayer, 5-min warm-up, 30-40 min of MVPA, 10-minute cool-down, and a brief discussion of the month’s health topic (e.g., proper hydration, injury prevention, myths about PA). To assess the intensity and quality of *promotora*-led PA classes, we used System for Observing Fitness Instruction Time in Group Exercise Classes (SOFIT-X), an observational tool to evaluate group exercise classes [27]. *Promotoras* recorded attendance at classes and called absent participants to encourage them to attend classes.

*Promotoras* conducted up to four motivational interviewing (MI) calls each year over the course of the 2-year intervention following guidelines by Resnicow and colleagues [28]. Calls included discussions of the participant’s engagement in MVPA, barriers to PA, personal values, and goal setting. Participants received monthly health handouts on various topics related to PA, and *promotoras* reinforced these topics at the end of each PA class. *Promotoras* were supported by the Physical Activity Specialist through regular in-person meetings, observations and feedback of classes, and booster trainings throughout the 24-month intervention.

Given the influence of the built environment on Latinas’ PA [29–32], *Faith in Action* also targeted environmental influences. *Promotoras* received training from Circulate San Diego, a local advocacy organization (www.circulatesd.org), to conduct walk audits and advocate for safe and accessible spaces to be active. *Promotoras* worked with churchgoers to identify projects to improve the built environment for PA at their church site and in the surrounding neighborhood. For example, participants identified sidewalk improvements, park clean-up projects, trail restoration, community gardens, and planting natural buffers between the church site and a trolley stop to increase safety.

Participants in the control condition received general cancer prevention information including colon, skin, breast, and cervical cancer conducted in the same manner as the PA intervention condition. *Promotoras* held 1 h group workshops each week promoting cancer prevention using the similar protocols outlined in the PA intervention condition. The *promotoras* were responsible for conducting MI calls on the same set of participants each month. The PA intervention and cancer screening conditions were designed to be equivalent in all respects except for content.

### Data collection and measures

Bilingual/bicultural research assistants blind to experimental condition collected data at baseline, 12- and 24-months. Data were collected at each church site from 2010 to 2016. At each data collection point, participants who were also blind to study condition attended two appointments. At the first appointment, research assistants assessed anthropometrics (height, weight, waist circumference), and fitted participants with an accelerometer to wear for 7 days. At the second appointment, participants turned in accelerometers and completed a survey that collected demographic, health, psychosocial, and neighborhood environment data. At each timepoint, participants received $25 for completing the evaluation protocol.

#### Accelerometer-assessed PA (primary outcome)

Participants were properly fitted with the hip worn GT3-X+ activity monitor (Actigraph, Pensacola, FL) prior to the wear period (defined as at least 12 h per day for 7 days). Valid data were defined as at least 5 days, including one weekend day, with ≥10 valid h per day. An invalid hour was defined as > 60 consecutive minutes of zero count values. Participants who did not meet the criteria for minimum wear time were asked to re-wear the device. Data were processed using the ActiLife software with each minute counted using Troiano 2008 cut-points, which define MVPA as 2020 counts per minute or more, light PA as 100-2019 counts per minute, and sedentary as 0-99 counts per minute [33]. Minutes of MVPA were used as a normally-distributed continuous variable.

#### Self-report PA (primary outcome)

The Global Physical Activity Questionnaire (GPAQ) assessed PA, including leisure-time, transportation, and occupation domains. The GPAQ has been validated against the accelerometer and has shown high reliability for vigorous PA among Latinas [34]. Total minutes/week of PA in each domain was computed using standard GPAQ protocol [35]. We classified participants as meeting national guidelines [36] if they reported ≥150 min/wk. of moderate PA, or ≥ 75 min/wk. of vigorous PA, or ≥ 600 MET-min of MVPA during combined leisure-time and transportation PA.

#### Anthropometric measures (secondary)

Trained research assistants weighed and measured women using standard procedures as previously described [21]. Body mass index (BMI) was calculated as weight [kg]/height [m^2^].

#### Demographics and health conditions (secondary)

The study questionnaires collected demographic information such as age, education (recoded as completed high school or not), employment status (recoded as employed or not), monthly household income (<$2000 vs. ≥$2000), country of birth (Mexico vs. USA or other foreign country), number of years living in the US, and marital status (married/living as married vs. single/non-partnered). Health conditions assessed included self-reported physician-diagnosed diabetes, arthritis, coronary heart disease, and cancer using questions from the Behavioral Risk Factor Surveillance System (BRFSS) 2011 [37].

#### Process evaluation

Process evaluation determined whether the intervention was delivered with fidelity, adhering to the overall planned dose. *Promotoras* completed weekly activity logs indicating intervention activities attempted or completed, which provided information on intervention dose (e.g., number of sessions held; number of participants). We also collected attendance sheets, call logs, and MI logs. The Intervention Coordinator conducted quality control checks with the *promotoras* to ensure they followed project protocols. We used SOFIT-X observations to assess the intensity and quality of *promotora*-led PA classes [27]. Briefly, SOFIT-X is a measure that can be used to reliably code participant posture, class context, and instructor behavior in adult group-exercise classes.

### Statistical analyses

All analyses were based on the intention-to-treat approach. Each outcome was examined using mixed effects models for normal outcomes (SAS Proc Mixed) or generalized linear mixed effects models for non-normal outcomes (SAS Proc Glimmix) to account for the three-level data structure of repeated measures within participants and participants nested within churches. For non-normal outcomes, appropriate error distribution and link functions were chosen according to the type of outcome. The primary outcome of the trial was MVPA (self-report and accelerometer assessed) and secondary outcomes were: light intensity activity, percent sedentary time, BMI, and waist circumference.

This paper describes results through a 24-month period and includes 12-month data. Models accounted for repeated measures over 12 and 24 months and adjusted for the baseline level of the outcome. Analyses used all available data; thus, if a participant had data missing at 12 or 24 months, analyses still included data at nonmissing time points. Terms in the model included a condition indicator (intervention vs control), time (12 vs 24 months), and the group by time interaction. If the interaction was not significant, the interaction term was dropped and the condition main effect was examined. All models adjusted for age (continuous), marital status (married or living as married vs single or no partner), employment (yes, no) and education (completed high school: yes, no). The covariates were selected a priori to improve precision of the estimates. All analyses were carried out at the .05 level of significance.

#### Dose-response

We assessed dose-response associations in the intervention condition only using number of MI calls completed each year and number of PA classes attended monthly as dose indicators. Based on the distributions, MI calls were dichotomized into 0-1 and 2-4 calls per year 1 and year 2. Similarly, class attendance was dichotomized into less than once a month and at least once a month for each year. Our dose-response analysis used similar models to those described above except that a dose-response indicator replaced the condition indicator.

## Results

**Fig. 1:**
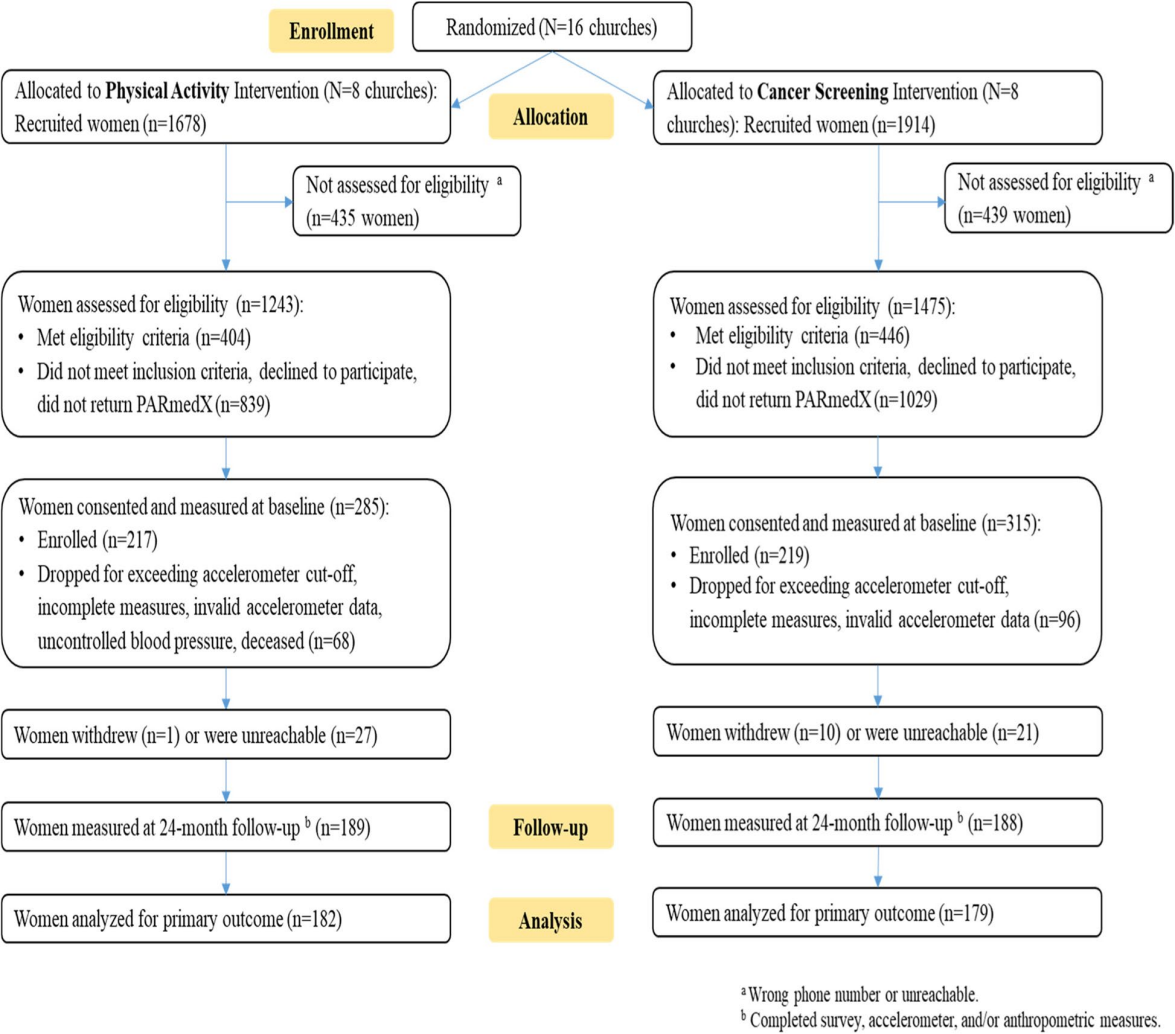
Consort flow chart: Fe en Accion, San Diego, CA

Figure 1 shows the Faith in Action 24-month CONSORT flowchart, which describes the recruitment and retention outcomes for this study. Of the 2718 individuals recruited and screened for the study, 436 were enrolled (*n* = 217 in intervention and *n* = 219 in cancer screening). Figure 1 demonstrates good cohort maintenance rate at 87% at 12 months and 86% at 24 months.

Additional file 1: Table 1 presents descriptive statistics of the sample, and Additional file 2: Table 2 provides descriptive statistics of the outcome variables.

### Outcomes analysis

Table 1 None of the time by condition interactions were significant, indicating no evidence that condition effects, if any, varied over time. Accelerometer-assessed MVPA was in the marginally significant range (*p* < .08). Overall, averaging across the two time periods, the intervention condition MVPA value was higher than the control condition (4.71 vs 4.59 log units). Self-report leisure time MVPA was significant (*p* < .005); the intervention condition had a higher adjusted mean compared to attention-control condition (3.20 vs 2.44 log units). The odds of meeting the MVPA recommendation from leisure and transportation domains was 80% higher in the intervention group compared to control (OR = 1.80, 95% CI: 1.21, 2.67, *p* < .004). There were no significant effects on light intensity activity, sedentary time, BMI, and waist circumference.

**Table 1:** Mixed effects models^a^ to evaluate intervention across 12 and 24 months post-baseline

**Outcomes**	Time X Condition Interaction *P*-value	Condition						
		Intervention		Control		Difference (Inter – Control)		
		Adj Mean	SE	Adj Mean	SE	Diff in adj means	*P*-value	Effect Size
**Primary**								
Accelerometer MVPA^b^	.81	4.71	0.05	4.59	0.05	0.12	.081	.18
Self report leisure time MVPA^b^	.37	3.29	0.20	2.44	0.22	0.85	.005	.30
**Secondary**								
Accelerometer light activity	.69	2342.2	29.3	2324.1	25.0	18.1	.64	.05
Accelerometer percent sedentary time	.71	75.1	0.30	75.4	0.26	− 0.3	.61	.09
BMI	.10	30.3	0.13	30.5	0.12	− 0.2	.33	.12
Waist circumference	.45	95.7	0.4	96.3	0.4	− 0.6	.27	.10
		Odds Ratio					*P*-value	
Meets MVPA recommendation from leisure & transport domains^c^		Estimate		95% CI				
Intervention vs Control	.75	1.80		1.21, 2.67			.004	

^a^Mixed effects or generalized linear mixed models were used to adjust for the clustering effects of churches and to account for repeated measures over M2 and M3. If the time by condition interaction term was not significant, the term was dropped and the condition main effect was tested. All analyses were adjusted for the baseline measure of the outcome, age, marital status, employment and education

^b^Negative binomial error distribution. Results are shown in logged units

^c^Binomial error distribution (Logistic model)

#### Dose response

At 12 months, about 54% of participants received 2-4 calls and 45% received 0-1 call. At 24 months, 59% received 2-4 calls and 40% received 0-1 call, which included only the last 12 months. When considering class attendance, 23% attended at least once a month during the first 12 months, and 12% attended at least once a month in the last 12 months. Table 2 examines dose-response findings for MI calls. Only self -report leisure time MVPA demonstrated a significant dose-response association (*p* < .003), where 2-4 calls had a higher adjusted mean than 0-1 calls (3.54 vs 3.01 log units).

**Table 2:** Dose-response^a^ for Motivational Interviewing calls in the PA condition across M2 and M3

**Outcomes**	**Motivational Interviewing calls**						
	2 – 4 calls/yr		0 – 1 calls/yr				
	Adj Mean	SE	Adj Mean	SE	Diff in adj means	*P*-value	Effect Size
**Primary**							
Accelerometer MVPA^b^	4.67	0.06	4.68	0.07	− 0.01	.89	.02
Self report leisure time MVPA^b^	3.54	0.20	3.01	0.23	+ 0.53	.003	.25
**Secondary**							
Accelerometer light activity	2324.1	38.0	2339.7	44.7	− 15.6	.81	.04
Accelerometer % sedentary time	75.3	0.4	75.2	0.5	+ 0.1	.81	.03
BMI	31.0	0.17	31.0	0.13	0	.81	.01
Waist circumference	96.6	0.65	97.0	0.56	− 0.40	.57	.06
	Odds Ratio					*P*-value	
Meets MVPA recommendation from leisure & transport domains^c^	Estimate		95% CI				
Intervention vs Control	1.43		0.90, 2.25			.13	

For BMI and waist circumference, models were rerun eliminating time points where women indicated they were pregnant during that period. However, no notable differences were found

^a^Mixed effects or generalized linear mixed models were used to adjust for the clustering effects of churches and to account for repeated measures over M2 and M3. All analyses were adjusted for the baseline measure of the outcome, age, marital status, employment and education

^b^Negative binomial error distribution. Results are shown in logged units

^c^Binomial error distribution (Logistic model)

Table 3 displays the results for class attendance. Participants who attended the PA classes at least once a month had higher accelerometer-assessed MVPA, higher leisure time MVPA, and more met the MVPA recommendations by self-report compared to those who attended the classes less than once a month.

**Table 3:** Dose-response^a^ for class attendance in the PA condition across M2 and M3

**Outcomes**	**Class attendance**						
	At least once a month		Less than once a month				
	Adj Mean	SE	Adj Mean	SE	Diff in adj means	*P*-value	Effect Size
**Primary**							
Accelerometer MVPA^b^	4.92	0.09	4.63	0.06	+ 0.29	.002	.41
Self report leisure time MVPA^b^	4.11	0.27	3.16	0.21	+ 0.95	.001	.39
**Secondary**							
Accelerometer light activity	2368.7	62.2	2322.7	28.8	+ 46.0	.50	.13
Accelerometer % sedentary time	74.7	0.65	75.4	0.30	− 0.7	.32	.19
BMI	30.7	0.20	31.0	0.17	− 0.3	.24	.15
Waist circumference	96.1	0.52	97.0	0.61	− 0.9	.30	.13
	Odds Ratio					*P*-value	
Meets MVPA recommendation from leisure & transport domains^c^	Estimate		95% CI				
Intervention vs Control	7.85		4.21, 14.6			<.001	

For BMI and waist circumference, models were rerun eliminating time points where women indicated they were pregnant during that period. However, no notable differences were found

^a^Mixed effects or generalized linear mixed models were used to adjust for the clustering effects of churches and to account for repeated measures over M2 and M3. All analyses were adjusted for the baseline measure of the outcome, age, marital status, employment and education

^b^Negative binomial error distribution. Results are shown in logged units

^c^Binomial error distribution (Logistic model)

## Discussion

Findings from *Faith in Action* showed participants in the PA condition reported more leisure time MVPA and were significantly more likely to report meeting the national PA guidelines 2 years following baseline compared to those in the attention-control condition. For accelerometer-assessed MVPA, there were no significant differences between the intervention and control 2 years after starting the intervention. There was a trend (*p* < .08) to indicate that participants in the PA condition were more likely to engage in MVPA, but this was not maintained following the significant effects founds at 12 months [38]. Further, there were no significant intervention effects at 24 months on secondary outcomes including light intensity activity, sedentary time, BMI, and waist circumference.

Participants who attended the PA classes at least once a month were more likely to engage in MVPA (objective and self-report) compared to those who attended less frequently. The impact of MI telephone calls on intervention participants’ PA was less clear. Participants in the PA condition who received 2-4 calls over the course of 12 months reported engaging in significantly more leisure time MVPA than those who completed 0-1 call. However, completion of MI calls was not related to more accelerometer-assessed MVPA. The mixed findings in the present study are similar to conclusions reported in systematic reviews evaluating the impact of MI on PA [39–41].

### Limitations and strengths

Given the intervention approaches used in *Faith in Action* and the inclusion of only Latinas, our findings are not generalizable to men or members of other racial/ethnic groups. Faith-based organization (FBO) leaders (i.e., pastors) were not directly involved in the implementation of program activities, which is proving to be an important factor in implementing and sustaining health promotion interventions in FBOs [42–44]. Although the intervention included organizational and environmental change strategies, it was not possible to evaluate the impact of these strategies on the primary outcomes due to the small number of churches that were randomized to each condition (8 churches per condition), limiting the power to detect potential differences. Lastly, fewer participants attended the PA classes on a regular basis in the second year compared to the first year which may explain, in part, the lower intervention effects found at 24-months in the accelerometer assessed MVPA, BMI, and waist circumference outcomes compared to the 12-month effects [38]. These data suggest that stronger engagement strategies are needed to assure greater attendance to program activities over long periods of time.

Although there were some limitations, our study had substantial strengths. This study was a church-based clustered randomized controlled trial that included an attention-control (both conditions included MI calls and were group-based) rather than no-treatment control condition, strengthening the internal validity. The current study adds to the limited PA intervention research that examined intervention effects beyond 12 months using self-report and device-based assessments of PA. Previous systematic reviews have called for the investigation in the impact of longer community-based PA randomized trials on behavioral outcomes and objective assessment of activity in community settings [45]. The limited number of studies that examine the long term impact of PA interventions in faith based settings have included non-Latino communities and findings have met with mixed results [46–48] To our knowledge, this is the first published randomized controlled trial in faith based settings that reports on the long term (> 15 months) PA outcomes among US Latinos.

## Conclusions and future directions

Many long term interventions are successful in initiating PA but may need to consider including strategies to overcome relapse and sustain PA following the initial behavior change [49]. Although significant intervention effects documented at 12 months were not maintained at 24 months on accelerometer-assessed PA, there was still evidence of longer-term benefits through higher self-reported leisure time PA at 2 years compared to baseline. *Faith in Action/Fe en Accion* was successful in helping inactive Latinas increase their PA by demonstrating safe ways to be active during the PA classes, helping participants set realistic goals through educational handouts distributed during the PA classes and MI calls, rewarding participation in PA through monthly raffles, and improving opportunities for PA in places where people live and worship. However, inactive individuals who successfully begin PA are at risk of lapse or relapse into inactivity [50, 51] As such, long term PA community interventions may benefit from including low cost strategies to support PA like activity trackers and apps which are increasingly integrating evidence-based behavioral strategies such as goal setting, reminders, feedback, and accountability [52, 53].

Enhancing the capacity in FBOs to implement and sustain multilevel PA interventions may augment the effectiveness and sustainability of PA programs in churches. Given their influential role, FBO leaders have the potential to influence the attitudes and health behaviors of churchgoers [54, 55]. Pastors are decision makers who can facilitate the successful implementation and maintenance of program activities in church settings. Thus, training FBO leaders at the start of an intervention with strategies to improve their own health, practical skills to motivate members to be active [56], and strategies to make FBOs’ environments more supportive of healthy behaviors might enhance the reach, effectiveness, and sustainability of PA programs.

PA interventions in FBO settings have the potential to address racial/ethnic disparities in PA. *Faith in Action* was a culturally tailored program that trained bilingual *promotoras* who are often members of their community to deliver a PA program in their community. In addition to using strategies that targeted individuals’ health beliefs and practices, *Faith in Action* targeted the larger context in which individuals live, thereby increasing access to PA opportunities and providing supportive environments for PA maintenance. While FBOs are promising settings for health promotion interventions, more research is needed on mechanisms of change as well as how to implement, sustain, and scale-up evidence-based programs in FBOs. Future studies are needed to test additional implementation strategies in Latino churches, particularly given the importance of the church for Latino communities.

## Supplementary Information

**Additional file 1: Table 1.** Characteristics of participants at baseline by study condition and overall. *Fe en Acción*, San Diego, CA.

**Additional file 2: Table 2.***Fe En Accion* Descriptive statistics outcomes and potentials mediators M1 – M3.

## Data Availability

The datasets used and/or analyzed during the current study are available from the corresponding author on reasonable request. Intervention materials are available at https://ebccp.cancercontrol.cancer.gov/index.do

## Abbreviations

FBO: Faith Based Organizations
PA: Physical Activity
MPVA: Moderate-to-vigorous physical activity
EBCCP: Evidence-Based Cancer Control Programs
US: United States
MI: Motivational Interviewing
GPAQ: Global Physical Activity Questionnaire
BRFSS: Behavioral Risk Factor Surveillance System
BMI: Body mass index.
