# Supplementary material for: Two-year outcomes of Faith in Action/Fe en Acción: a randomized controlled trial of physical activity promotion in Latinas
Source: Int J Behav Nutr Phys Act. 2022 Jul 30;19:97. doi: 10.1186/s12966-022-01329-6 (PMC9338625; doi:10.1186/s12966-022-01329-6)
Supplement: Supplementary file 1 — Additional file 1: Table 1. Characteristics of participants at baseline by study condition and overall. Fe en Acción, San Diego, CA. [file 12966_2022_1329_MOESM1_ESM.docx]

**Additional file 1: Table 1.** Characteristics of participants at baseline by study condition and overall. *Fe en Acción*, San Diego, CA.

|  | Physical Activity  (n=217) | Attention Control (n=219) | Overall  (N=436) |
| --- | --- | --- | --- |
| *Demographics* | Mean (SD) | Mean (SD) | Mean (SD) |
| Age, years | 44.5 (9.8) | 44.4 (9.4) | 44.4 (9.6) |
| Residence in the US, years | 22.1 (11.2) | 19.8 (9.2) | 21.0 (10.3) |
|  |  |  |  |
|  | n (%) | n (%) | n (%) |
| Born in Mexico ^a^ | 193 (88.9) | 201 (92.6) | 394 (90.8) |
| Married/living as married | 168 (78.1) | 166 (76.5) | 334 (77.3) |
| Employed ^b^ | 147 (68.7) | 138 (63.0) | 285 (65.8) |
| Monthly household income < $2000 | 115 (56.1) ^c^ | 121 (60.5) ^d^ | 236 (58.3) ^e^ |
| Less than high school completed | 116 (53.7) | 122 (56.0) | 238 (54.8) |
|  |  |  |  |
| *Physician-diagnosed health conditions* |  |  |  |
| Diabetes | 27 (12.4) | 23 (10.5) | 50 (11.5) |
| Arthritis | 24 (11.3) | 22 (10.3) | 46 (10.8) |
| Heart disease | 9 (4.2) | 8 (3.8) | 17 (4.0) |
| Cancer | 9 (4.3) | 7 (3.3) | 16 (3.8) |
|  |  |  |  |
| *Anthropometrics* | Mean (SD) | Mean (SD) | Mean (SD) |
| BMI, kg/m^2^ | 30.8 (6.6) | 29.9 (5.8) | 30.3 (6.2) |
| Waist circumference, cm | 95.8 (15.0) | 94.1 (14.3) | 94.9 (14.7) |
|  |  |  |  |
| *Accelerometer-based PA* |  |  |  |
| MVPA (primary aim), min/week | 98.8 (61.5) | 107.6 (65.8) | 103.2 (63.8) |
| Moderate PA, min/week | 98.1 (61.0) | 106.2 (64.7) | 102.2 (63.0) |
| Vigorous PA, min/week | 0.7 (3.6) | 1.4 (6.1) | 1.1 (5.0) |
|  |  |  |  |
| *Self-report PA* |  |  |  |
| Leisure-time MVPA, min/week | 71.2 (121.1) | 64.0 (104.1) | 67.6 (112.8) |
| Moderate leisure-time PA, min/week | 51.4 (97.3) | 48.8 (79.9) | 50.1 (88.9) |
| Vigorous leisure-time PA, min/week | 19.8 (62.5) | 15.2 (53.8) | 17.5 (58.3) |
| Transportation PA, min/week | 66.1 (198.3) | 49.7 (109.6) | 57.9 (160.2) |
|  |  |  |  |
|  | n (%) | n (%) | n (%) |
| Meets MVPA recommendations ^g^ | 71 (32.7) | 69 (31.8) | 140 (32.3) |

*Notes*: PA: physical activity; BMI=body mass index; MVPA=moderate-to- vigorous physical activity; PA=physical activity; SD= Standard deviation

^a^ Remainder include those born in the US or another foreign country.

^b^ Includes those employed full-time, part-time, seasonally, or self-employed.

^c^ Missing n=12.

^d^ Missing n=19.

^e^ Missing n=31.

^f^ Out of total wear time.

^g^ Reported ≥150 min/wk of moderate aerobic activity, or ≥75 min/wk of vigorous aerobic activity, or an equivalent combination of both, in the leisure-time and/or transportation PA domains.
