# Supplementary material for: Two-year outcomes of Faith in Action/Fe en Acción: a randomized controlled trial of physical activity promotion in Latinas
Source: Int J Behav Nutr Phys Act. 2022 Jul 30;19:97. doi: 10.1186/s12966-022-01329-6 (PMC9338625; doi:10.1186/s12966-022-01329-6)
Supplement: Supplementary file 2 — Additional file 2: Table 2.Fe En Accion Descriptive statistics outcomes and potentials mediators M1 – M3. [file 12966_2022_1329_MOESM2_ESM.docx]

| **Appendix Table 2**: Fe En Accion Descriptive statistics outcomes and potentials mediators M1 – M3 | | | | | | | |
| --- | --- | --- | --- | --- | --- | --- | --- |
|  | Time | Condition | | | | | |
|  |  | Intervention | | | Control | | |
| **Primary Outcomes** |  | N | Mean | SD | N | Mean | SD |
| Actilife MVPA (min/week) | M1 | 217 | 98.8 | 61.5 | 219 | 107.6 | 65.8 |
|  | M2 | 183 | 144.4 | 117.9 | 186 | 129.9 | 125.6 |
|  | M3 | 187 | 133.9 | 113.2 | 182 | 124.7 | 119.0 |
|  |  |  |  |  |  |  |  |
| Leisure time MVPA self report | M1 | 217 | 71.2 | 121.0 | 219 | 64.0 | 104.1 |
| (min/week) | M2 | 187 | 138.2 | 178.0 | 188 | 91.7 | 134.4 |
|  | M3 | 187 | 136.9 | 197.7 | 184 | 88.8 | 116.6 |
|  |  |  |  |  |  |  |  |
|  |  | N | n | % | N | n | % |
| Meets MVPA recommendations from | M1 | 217 | 71 | 32.7 | 217 | 69 | 31.8 |
| leisure and transport domains | M2 | 186 | 99 | 53.2 | 187 | 75 | 40.1 |
|  | M3 | 187 | 96 | 51.3 | 183 | 73 | 39.9 |
|  |  |  |  |  |  |  |  |
| Actilife Light activity (min/week) | M1 | 217 | 2308.2 | 570.9 | 219 | 2303.7 | 604.8 |
|  | M2 | 183 | 2356.2 | 615.5 | 186 | 2360.7 | 600.7 |
|  | M3 | 187 | 2317.7 | 587.5 | 182 | 2294.3 | 568.9 |
|  |  |  |  |  |  |  |  |
| Actilife Percent sedentary time | M1 | 217 | 75.8 | 5.9 | 219 | 75.8 | 6.3 |
|  | M2 | 183 | 75.0 | 6.6 | 186 | 74.9 | 6.5 |
|  | M3 | 187 | 75.5 | 6.3 | 182 | 75.7 | 6.2 |
|  |  |  |  |  |  |  |  |
| BMI | M1 | 216 | 30.8 | 6.6 | 218 | 29.9 | 5.8 |
|  | M2 | 187 | 30.7 | 6.4 | 190 | 30.1 | 6.1 |
|  | M3 | 185 | 31.2 | 6.8 | 187 | 29.7 | 5.9 |
|  |  |  |  |  |  |  |  |
| Waist circumference (cm) | M1 | 216 | 95.7 | 15.0 | 218 | 94.1 | 14.3 |
|  | M2 | 187 | 96.2 | 14.3 | 190 | 95.7 | 15.3 |
|  | M3 | 187 | 97.1 | 15.3 | 188 | 94.9 | 14.3 |
|  |  |  |  |  |  |  |  |
